# Supplementary material for: Gorse (Ulex europeaus) wastes with 5,6-dimethyl benzimidazole supplementation can support growth of vitamin B12 producing commensal gut microbes
Source: PLoS One. 2024 Feb 29;19(2):e0290052. doi: 10.1371/journal.pone.0290052 (PMC10903898; doi:10.1371/journal.pone.0290052)
Supplement: S1 File — All supplementary files and information is provided in this document. (PDF) [file pone.0290052.s002.pdf]

Supplementary Material for: Vitamin B_{12} synthesis in three commensal gut microbes and their application in valorising Gorse (*Ulex europeaus*) wastes.

Ajay Iyer[1,3], Eva C. Soto-Martin[2], Gary Cameron[1], Charles S. Bestwick[1], Sylvia H. Duncan[1], and Wendy R. Russell*[1]

[1]University of Aberdeen, Rowett Institute, Aberdeen AB25 2ZD, Scotland
[2]University of Aberdeen, IMS, Aberdeen AB25 2ZD, Scotland
[3]Teagasc, Fermoy, Co Cork, Ireland.

1 Supplementary texts

Additional details and codes may be found in the OSF repository: https://osf.io/3yb2r/

1.1 M2GSC recipe

Bacto™ Casitone (10 g·L^{-1}), Bacto™ Yeast Extract (2.5 g·L^{-1}), sodium bicarbonate ($NaHCO_3$, 4.0 g·L^{-1}), glucose (2 g·L^{-1}), soluble starch (2 g·L^{-1}), cellobiose (2 g·L^{-1}), clarified rumen fluid (100 mL·L^{-1}), cysteine HCl (1 g·L^{-1}), dipotassium hydrogen phosphate (K_2HPO_4, 0.4 g·L^{-1}), potassium dihydrogen phosphate (K_2HPO_4, 0.4 g·L^{-1}), ammonium sulphate ($(NH_4)_2SO_4$, 0.8 g·L^{-1}), sodium chloride (NaCl, 0.8 g·L^{-1}), magnesium sulphate ($MgSO_4$, 80 mg·L^{-1}), calcium chloride ($CaCl_2$, 80 mg·L^{-1}) and resazurin (1 µg·L^{-1}).

1.2 Chemically defined medium (CDM)

Nitrogen sources (in levo-chirality): alanine (6.2 mg·L^{-1}), arginine (7.3 mg·L^{-1}), aspartate (4.1 mg·L^{-1}), aspartic acid (5.4 mg·L^{-1}), glutamine (4.2 mg·L^{-1}), glutamic acid (7.1 mg·L^{-1}), glycine (4.1 mg·L^{-1}), histidine (2.5 mg·L^{-1}), isoleucine (6.7 mg·L^{-1}), leucine (10.4 mg·L^{-1}), lysine (6.4 mg·L^{-1}), methionine (2.9 mg·L^{-1}), phenylalanine (5.3 mg·L^{-1}), proline (3.8 mg·L^{-1}), serine (4.9 mg·L^{-1}), threonine (4.8 mg·L^{-1}), tryptophan (1.9 mg·L^{-1}), valine (6.3 mg·L^{-1}), cysteine (10 mg·L^{-1}).

*email: w.russell@abdn.ac.uk

Carbon sources (in dextro-chirality): glucose ($2\,g{\cdot}L^{-1}$), galactose ($2\,g{\cdot}L^{-1}$), cellobiose ($2\,g{\cdot}L^{-1}$) and potassium hydrogen carbonate ($KHCO_3$, $40\,mg{\cdot}L^{-1}$).

Mineral sources: dipotassium hydrogen phosphate (K_2HPO_4, $0.2\,mg{\cdot}L^{-1}$), ammonium sulphate ($(NH_4)_2SO_4$, $4\,mg{\cdot}L^{-1}$), sodium chloride (NaCl, $4\,mg{\cdot}L^{-1}$), magnesium sulphate, heptahydrate ($MgSO_4 \cdot 8\,H_2O$, $0.4\,mg{\cdot}L^{-1}$), calcium chloride ($CaCl_2$, $0.4\,mg{\cdot}L^{-1}$) and potassium dihydrogen phosphate (KH_2PO_4, $2\,mg{\cdot}L^{-1}$), ferrous sulphate heptahydrate ($FeSO_4 \cdot 7\,H_2O$, $21\,mg{\cdot}L^{-1}$), zinc sulphate heptahydrate ($ZnSO_4 \cdot 7\,H_2O$, $1.8\,mg{\cdot}L^{-1}$), boric acid (H_3BO_3, $5\,mg{\cdot}L^{-1}$), cobalt chloride hexahydrate ($CoCl_2 \cdot 6\,H_2O$, $10\,mg{\cdot}L^{-1}$), nickel chloride hexahydrate ($NiCl_2 \cdot 6\,H_2O$, $0.1\,mg{\cdot}L^{-1}$), copper chloride dihydrate ($CuCl_2 \cdot 2\,H_2O$, $0.1\,mg{\cdot}L^{-1}$), manganese chloride tetrahydrate ($MnCl_2 \cdot 4\,H_2O$, $0.7\,mg{\cdot}L^{-1}$), sodium molybdate dihydrate ($Na_2MoO_4 \cdot 2\,H_2O$, $0.5\,mg{\cdot}L^{-1}$), sodium selenite pentahydrate ($Na_2SeO_3 \cdot 5\,H_2O$, $0.1\,mg{\cdot}L^{-1}$), sodium tungstate dihydrate ($Na_2WO_4 \cdot 2\,H_2O$, $0.1\,mg{\cdot}L^{-1}$).

Others: 5,6-dimethylbenzimidazole (DMB, $20\,mg{\cdot}L^{-1}$), pyridoxine HCl ($1\,ng{\cdot}L^{-1}$), pyridoxal HCl ($1\,ng{\cdot}L^{-1}$), pyridoxamine HCl ($1\,ng{\cdot}L^{-1}$), thiamine HCl ($0.5\,ng{\cdot}L^{-1}$), Ca-D-pantothenate (B5, $0.5\,ng{\cdot}L^{-1}$), pantetheine ($0.5\,ng{\cdot}L^{-1}$), nicotinic acid ($0.5\,ng{\cdot}L^{-1}$), nicotinamide ($0.5\,ng{\cdot}L^{-1}$), riboflavin ($0.5\,ng{\cdot}L^{-1}$), folic acid ($0.5\,ng{\cdot}L^{-1}$), p-amino benzoic acid ($0.5\,ng{\cdot}L^{-1}$), menadione ($0.1\,ng{\cdot}L^{-1}$), lipoic acid ($0.5\,ng{\cdot}L^{-1}$), pimelic acid ($0.2\,ng{\cdot}L^{-1}$), biotin ($0.2\,ng{\cdot}L^{-1}$).

Nucleotides: adenine ($10\,mg{\cdot}L^{-1}$), cytosine ($10\,mg{\cdot}L^{-1}$), thymine ($10\,mg{\cdot}L^{-1}$), xanthine ($10\,mg{\cdot}L^{-1}$), guanine ($10\,mg{\cdot}L^{-1}$), uracil ($10\,mg{\cdot}L^{-1}$), orotic acid ($5\,mg{\cdot}L^{-1}$) and thymidine ($10\,mg{\cdot}L^{-1}$).

1.3 LC/MS conditions for B_{12} estimation

Liquid chromatography was performed using An ACE Excel 3 µ C_{18} (150 mm × 2.1 mm) column maintained at 50 °C. Elution was performed across a gradient using solvent A (0.1% $v{\cdot}v^{-1}$ formic acid) and solvent B (methanol). The complete programme lasted 10 min at a flow rate of $200\,\mu L{\cdot}min^{-1}$ with a sample/standard injection volume of 5 µL. The column was maintained at 90:10:: Solvent A: Solvent B which was rapidly raised to 0:100::Solvent A:Solvent B at 4 min and maintained for 2 min. This was followed by a rapid drop to 90:10::Solvent A: Solvent B and maintained until the final 10 min runtime.

The mass spectrophotometry setup was performed using positive ion electrospray at 4 kV. Capillary pressure was 375 °C and collision pressure was 1.8 mTorr. Q1/Q3 peak width was 0.7. Approximate retention times (min) were: hydroxycobalamin (5.75), methyl-cobalamin (6.95), cyanocobalamin (6.25) and adenosylcobalamin (6.61). Calibration was performed using a quadratic transformation with $\frac{1}{x^2}$ weighting.

2 Supplementary figure

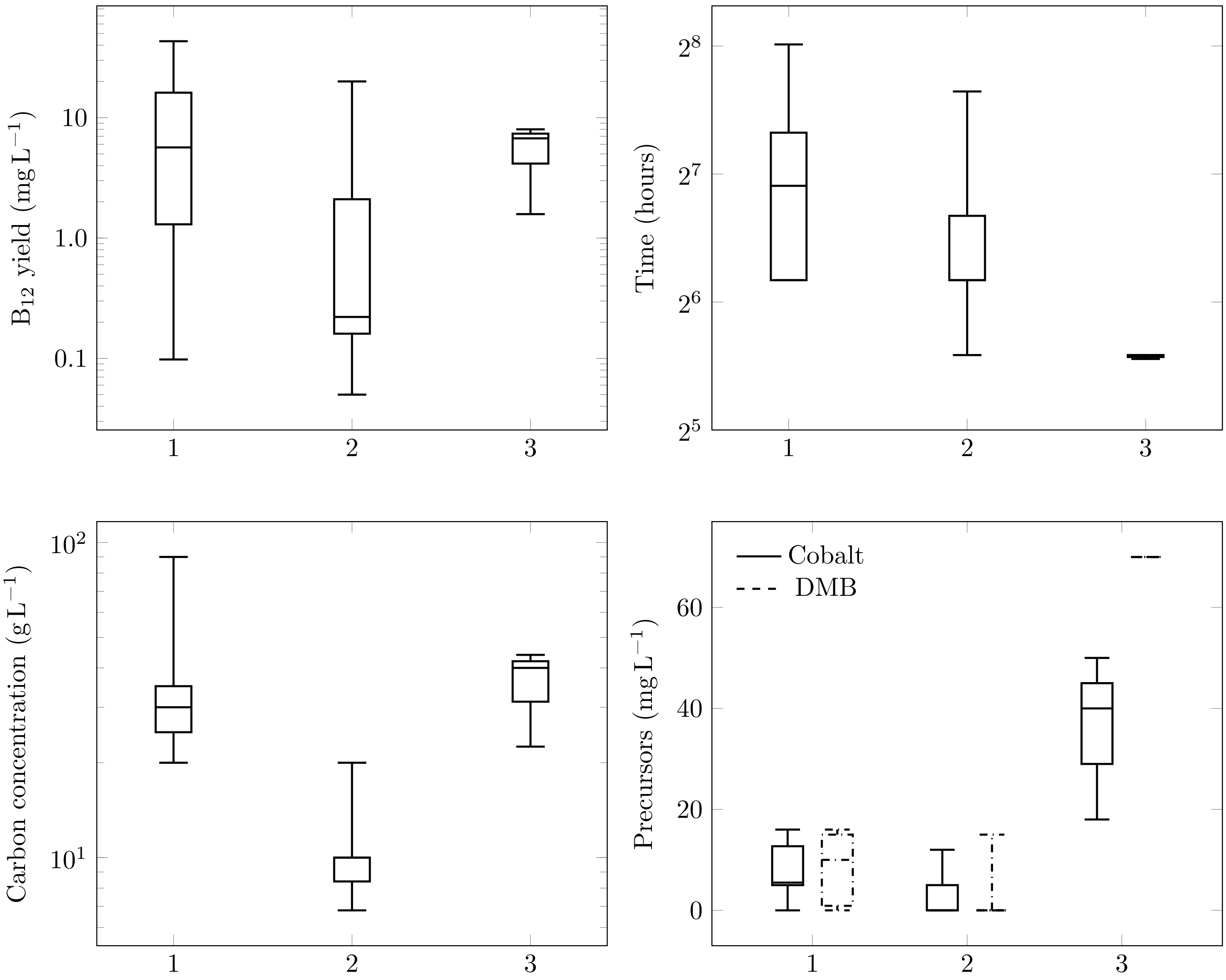

Supplementary Figure 1: Median levels of B_{12} yield, carbon source, precursors, and fermentation time in the k-means groups.

3 Supplementary table

Table 1: B_{12} production in previous literature. Data used for PCA analysis shown in Figure 3 in main document.

**Medium**	**DMB**	**Cobalt**	**B_{12}**	**Carbon**	**Time**	**Ref**
Casio peptone medium	70	18	6.73	40	48	[1]
Cereal matrix medium	15	5	1.5	20	144	[2]
Cheese propionic acid medium	0	0	0.124	6.8	108	[3]
Cheese whey + yeast extract	0	0	0.12	6.8	108	[3]
Cheese whey + yeast extract	5	5	20	7	168	[4, 5, 6]
Cornsteep liquor	0.9	16	12.5	41	132	[7]
Cornsteep liquor	0.9	12.7	21.5	56	84	[8]
Cornsteep liquor	0.9	5	18.2	35	160	[9]
Cornsteep liquor	0.9	12.7	35.2	20	72	[10]
Cornsteep liquor	0.9	12.7	43.5	30	258	[11]
Cornsteep liquor	14.6	5	2.06	90	72	[12]
Lactate Media	0	5	0.05	9	200	[13]
Lactate Media	0	5	0.16	9	200	[13]
Lactate Media	0	0	0.161	10	72	[14]
Lactate Media	0	0	0.227	10	72	[14]
Lactate Media	0	0	0.215	10	72	[14]
Lactate Media	0	0	0.681	10	72	[14]
Lactate Media	0	0	0.188	10	72	[14]
Lactate Media	0	0	0.168	10	72	[14]
Lactate Media	0	0	0.211	10	72	[14]
Lactate Media	0	0	0.221	10	72	[14]
pABA Medium	15	5	0.625	20	168	[15]
Peptone Medium	15	10	5.53	10	96	[16]
Peptone Medium	15	10	3.81	10	96	[16]
Peptone Medium	16	2	3.039	20	168	[17]
Soya Medium	15	15	0.528	20	100	[18]
Soya Medium	70	40	1.58	44	48	[19]
Spent media	0	0	0.95	10	72	[20]
Sunflower medium	15	5.5	1.6	40	160	[21]
Tofu waste	0	12	3.2	10	48	[22]
Whey permeate media	0	0	2.5	7.8	72	[23]
yeast extract	0	0	0.06	20	72	[24]
yeast extract	0	0	1.68	10	144	[25]
yeast extract	0	10	0.087	20	48	[26]
yeast extract	0	0	13.9	25	120	[27]
yeast extract	0.9	12.7	42.5	35	84	[28]
yeast extract	70	50	8	22.5	47	[29]
yeast extract	14.6	5	2.6	7.8	72	[30]
yeast extract	14.6	5	1.7	7.8	72	[30]
yeast extract	15	5.5	1.3	36	160	[21]
Yeast extract + lactate	15	5	0.223	32.8	120	[31]
Yeast extract + lactate	15	5	0.205	32.8	120	[31]
Yeast extract + lactate	15	5	0.098	32.8	120	[31]
Yeast extract + lactate	10	10	16.13	30	72	[32]
Yeast extract + lactate	10	10	10.93	30	72	[32]
Yeast extract + lactate	10	10	5.66	30	72	[32]
Yeast extract + lactate	10	10	12.06	30	72	[32]

Time expressed in h.
Carbohydrates (carbon) expressed in $g \cdot L^{-1}$.
Cobalt and 5,6-dimethylbenzimidazole (DMB) expressed in $mg \cdot L^{-1}$.

References

1. Miyano Ki, Ye K, Shimizu K. Improvement of vitamin B_{12} fermentation by reducing the inhibitory metabolites by cell recycle system and a mixed culture. Biochemical Engineering Journal. 2000;6(3):207–214. doi:10.1016/S1369-703X(00)00089-9.

2. Chamlagain B, Sugito TA, Deptula P, Edelmann M, Kariluoto S, Varmanen P, et al. *In situ* production of active vitamin B_{12} in cereal matrices using *Propionibacterium freudenreichii*. Food Science & Nutrition. 2018;6(1):67–76. doi:10.1002/fsn3.528.

3. Deptula P, Chamlagain B, Edelmann M, Sangsuwan P, Nyman TA, Savijoki K, et al. Food-Like Growth Conditions Support Production of Active Vitamin B_{12} by *Propionibacterium freudenreichii* 2067 without DMBI, the Lower Ligand Base, or Cobalt Supplementation. Frontiers in Microbiology. 2017;8. doi:10.3389/fmicb.2017.00368.

4. Bullerman LB, Berry EC. Use of Cheese Whey for Vitamin B_{12} Production: I. Whey Solids and Yeast Extract Levels. Applied Microbiology. 1966;14(3):353–355. doi:10.1128/am.14.3.353-355.1966.

5. Berry EC, Bullerman LB. Use of Cheese Whey for Vitamin B_{12} Production: II. Cobalt, Precursor, and Aeration Levels. Applied Microbiology. 1966;14(3):356–357. doi:10.1128/am.14.3.356-357.1966.

6. Bullerman LB, Berry EC. Use of Cheese Whey for Vitamin B_{12} Production: III. Growth Studies and Dry-Weight Activity. Applied Microbiology. 1966;14(3):358–360. doi:10.1128/am.14.3.358-360.1966.

7. Zhang Y, Li X, Wang Z, Wang Y, Ma Y, Su Z. Metabolic Flux Analysis of Simultaneous Production of Vitamin B_{12} and Propionic Acid in a Coupled Fermentation Process by *Propionibacterium freudenreichii*. Applied Biochemistry and Biotechnology. 2021;193(10):3045–3061. doi:10.1007/s12010-021-03584-y.

8. Wang P, Wang Y, Su Z. Improvement of Adenosylcobalamin Production by Metabolic Control Strategy in *Propionibacterium freudenreichii*. Applied Biochemistry and Biotechnology. 2012;167(1):62–72. doi:10.1007/s12010-012-9654-3.

9. Wang P, Wang Y, Liu Y, Shi H, Su Z. Novel *in situ* product removal technique for simultaneous production of propionic acid and vitamin B_{12} by expanded bed adsorption bioreactor. Bioresource Technology. 2012;104:652–659. doi:10.1016/j.biortech.2011.10.047.

10. Wang P, Jiao Y, Liu S. Novel fermentation process strengthening strategy for production of propionic acid and vitamin B_{12} by *Propionibacterium freudenreichii*. Journal of Industrial Microbiology and Biotechnology. 2014;41(12):1811–1815. doi:10.1007/s10295-014-1513-5.

11. Wang P, Shen C, Li L, Guo J, Cong Q, Lu J. Simultaneous production of propionic acid and vitamin B_{12} from corn stalk hydrolysates by *Propionibacterium freudenreichii* in an expanded bed adsorption bioreactor. Preparative Biochemistry & Biotechnology. 2020;50(8):763–767. doi:10.1080/10826068.2020.1734942.

12. Calvillo Ã, Pellicer T, Carnicer M, Planas A. Developing a single-stage continuous process strategy for vitamin B_{12} production with *Propionibacterium freudenreichii.* Microbial Cell Factories. 2023;22(1):26. doi:10.1186/s12934-023-02029-x.

13. Dank A, Biel G, Abee T, Smid EJ. Microaerobic metabolism of lactate and propionate enhances vitamin B_{12} production in *Propionibacterium freudenreichii.* Microbial Cell Factories. 2022;21(1):225. doi:10.1186/s12934-022-01945-8.

14. Van Wyk J, Witthuhn RC, Britz TJ. Optimisation of vitamin B_{12} and folate production by Propionibacterium freudenreichii strains in kefir. International Dairy Journal. 2011;21(2):69–74. doi:10.1016/j.idairyj.2010.09.004.

15. Hugenschmidt S, Schwenninger SM, Lacroix C. Concurrent high production of natural folate and vitamin B_{12} using a co-culture process with *Lactobacillus plantarum* SM39 and Propionibacterium freudenreichii DF13. Process Biochemistry. 2011;46(5):1063–1070. doi:10.1016/j.procbio.2011.01.021.

16. Khadiga AT, Mashhoor WW, Sohair A, Sharaf NMA. Production of Vitamin B_{12} by *Propionibacterium freudenreichii* and *Bacillus megaterium.* J Agric Sci Mansoura Univ. 2005;30(7):4149–4162.

17. Kośmider A, Białas W, Kubiak P, Drożdżyńska A, Czaczyk K. Vitamin B_{12} production from crude glycerol by *Propionibacterium freudenreichii* ssp. *shermanii*: Optimization of medium composition through statistical experimental designs. Bioresource Technology. 2012;105:128–133. doi:10.1016/j.biortech.2011.11.074.

18. de Assis DA, Matte C, Aschidamini B, Rodrigues E, Záchia Ayub MA. Biosynthesis of vitamin B_{12} by *Propionibacterium freudenreichii* subsp. *shermanii* ATCC 13673 using liquid acid protein residue of soybean as culture medium. Biotechnology Progress. 2020;36(5). doi:10.1002/btpr.3011.

19. Tanaka Y, Kasahara K, Izawa M, Ochi K. Applicability of ribosome engineering to vitamin B_{12} production by *Propionibacterium shermanii.* Bioscience, Biotechnology, and Biochemistry. 2017;81(8):1636–1641. doi:10.1080/09168451.2017.1329619.

20. Gardner N, Champagne CP. Production of *Propionibacterium shermanii* biomass and vitamin B_{12} on spent media. Journal of Applied Microbiology. 2005;99(5):1236–1245. doi:10.1111/j.1365-2672.2005.02696.x.

21. Hajfarajollah H, Mokhtarani B, Mortaheb H, Afaghi A. Vitamin B_{12} biosynthesis over waste frying sunflower oil as a cost effective and renewable substrate. Journal of Food Science and Technology. 2014;doi:10.1007/s13197-014-1383-x.

22. Yu Y, Zhu X, Shen Y, Yao H, Wang P, Ye K, et al. Enhancing the vitamin B_{12} production and growth of *Propionibacterium freudenreichii* in tofu wastewater via a light-induced vitamin B_{12} riboswitch. Applied Microbiology and Biotechnology. 2015;99(24):10481–10488. doi:10.1007/s00253-015-6958-6.

23. Hugenschmidt S, Schwenninger SM, Gnehm N, Lacroix C. Screening of a natural biodiversity of lactic and propionic acid bacteria for folate and vitamin B_{12} production in supplemented whey permeate. International Dairy Journal. 2010;20(12):852–857. doi:10.1016/j.idairyj.2010.05.005.

24. Bernhardt C, Zhu X, Schütz D, Fischer M, Bisping B. Cobalamin is produced by *Acetobacter pasteurianus* DSM 3509. Applied Microbiology and Biotechnology. 2019;103(9):3875–3885. doi:10.1007/s00253-019-09704-3.

25. Piao Y, Yamashita M, Kawaraichi N, Asegawa R, Ono H, Murooka Y. Production of vitamin B_{12} in genetically engineered *Propionibacterium freudenreichii*. Journal of Bioscience and Bioengineering. 2004;98(3):167–173. doi:10.1016/S1389-1723(04)00261-0.

26. Pillai VV, Prakash G, Lali AM. Growth engineering of *Propionibacterium freudenreichii shermanii* for organic acids and other value-added products formation. Preparative Biochemistry & Biotechnology. 2018;48(1):6–12. doi:10.1080/10826068.2017.1381619.

27. Piwowarek K, Lipińska E, Hać-Szymańczuk E, Bzducha-Wróbel A, Synowiec A. Research on the ability of propionic acid and vitamin B_{12} biosynthesis by *Propionibacterium freudenreichii* strain T82. Antonie van Leeuwenhoek. 2018;111(6):921–932. doi:10.1007/s10482-017-0991-7.

28. Wang P, Zhang Z, Jiao Y, Liu S, Wang Y. Improved propionic acid and 5,6- dimethylbenzimidazole control strategy for vitamin B_{12} fermentation by *Propionibacterium freudenreichii*. Journal of Biotechnology. 2015;193:123–129. doi:10.1016/j.jbiotec.2014.11.019.

29. Ye K, Shijo M, Jin S, Shimizu K. Efficient production of vitamin B_{12} from propionic acid bacteria under periodic variation of dissolved oxygen concentration. Journal of Fermentation and Bioengineering. 1996;82(5):484–491. doi:10.1016/S0922-338X(97)86988-7.

30. Chamlagain B, Deptula P, Edelmann M, Kariluoto S, Grattepanche F, Lacroix C, et al. Effect of the lower ligand precursors on vitamin B_{12} production by food-grade Propionibacteria. LWT - Food Science and Technology. 2016;72:117–124. doi:10.1016/j.lwt.2016.04.023.

31. Marwaha SS, Sethi RP, Kennedy JF. Influence of 5,6-dimethylbenzimidazole (DMB) on vitamin B_{12} biosynthesis by strains of Propionibacterium. Enzyme and Microbial Technology. 1983;5(5):361–364. doi:10.1016/0141-0229(83)90008-X.

32. Thirupathaiah Y, Swarupa Rani C, Sudhakara Reddy M, Venkateswar Rao L. Effect of chemical and microbial vitamin B_{12} analogues on production of vitamin B_{12}. World Journal of Microbiology and Biotechnology. 2012;28(5):2267–2271. doi:10.1007/s11274-012-1011-8.
